# Highly informative marker sets consisting of genes with low individual degree of differential expression

**DOI:** 10.1038/srep14967

**Published:** 2015-10-08

**Authors:** V. V. Galatenko, M. Yu. Shkurnikov, T. R. Samatov, A. V. Galatenko, I. A. Mityakina, A. D. Kaprin, U. Schumacher, A. G. Tonevitsky

**Affiliations:** 1 Moscow State University, Leninskie Gory, 119991 Moscow, Russia; 2 SRC Bioclinicum, Ugreshskaya str 2/85, 115088 Moscow, Russia; 3 P. Hertsen Moscow Oncology Research Institute, National Center of Medical Radiological Research, 3 Second Botkinsky Lane, Moscow, 125284, Russia; 4 Moscow State University of Mechanical Engineering, Bolshaya Semenovskaya str 38, 107023 Moscow, Russia; 5 Department of Anatomy and Experimental Morphology, University Cancer Center, University Medical Center Hamburg-Eppendorf, Martinistr. 52, Hamburg D-20246, Germany

## Abstract

Genes with significant differential expression are traditionally used to reveal the genetic background underlying phenotypic differences between cancer cells. We hypothesized that informative marker sets can be obtained by combining genes with a relatively low degree of individual differential expression. We developed a method for construction of highly informative gene combinations aimed at the maximization of the cumulative informative power and identified sets of 2–5 genes efficiently predicting recurrence for ER-positive breast cancer patients. The gene combinations constructed on the basis of microarray data were successfully applied to data acquired by RNA-seq. The developed method provides the basis for the generation of highly efficient prognostic and predictive gene signatures for cancer and other diseases. The identified gene sets can potentially reveal novel essential segments of gene interaction networks and pathways implied in cancer progression.

Transcriptomic analysis is an important tool for both theoretical research and clinical applications, including diagnosis, prognosis of disease and the optimal therapy choice^1^,^2^,^3^,^4^. However, it should be noted that for both theoretical research and clinical applications transcripts with the highest individual informative power are commonly used, more specifically the ones having the best differential expression characteristics including the lowest p-values and the highest fold-changes. This approach is used not only for the analysis related to individual transcriptomic markers, but also for the analysis dealing with combinations of transcripts, in particular, with gene signatures. Gene signatures are traditionally constructed by combining top N genes with respect to a certain measure of gene individual informative power, without any attempts to maximize the cumulative informative power of a gene combination. The top-N approach was used for the construction of such gene signatures, as Oncotype DX^5^, MammaPrint^6^, PAM50^7^. The N value (number of used genes) is typically less than a few hundreds and often comprises several dozens: 16 prognostic (and 5 control/reference) genes for OncotypeDX, 70 for MammaPrint, 50 for PAM50. Thus the use of top-N approach for gene signature construction automatically excludes all genes with a relatively low individual informative power from the scope of the analysis.

At the same time, as the synthetic example in Fig. 1 shows, genes with low individual informative power can make a significant contribution into the cumulative informative power of transcripts combination. In this synthetic example gene 1 has a high degree of differential expression, but does not provide the separation of groups. Gene 2 has a low degree of differential expression. However, the pair consisting of these genes provides ideal separation of groups confirming a significant contribution of gene 2 into the cumulative informative power of the pair.

We hypothesized that genes with low degree of differential expression could also work efficiently for experimental datasets. One of the well-known approaches to the utilization of expression data for genes with low individual informative power is to analyze gene expression correlations^8^,^9^. However, the fact that certain genes have highly correlated expression for one group of samples and low correlated expression for another group, reveals general differences of transcriptomes between groups of samples, but does not lead to the reliable conclusion about the attribution of a given individual sample to one of these groups.

In this work we constructed the classifiers that attributed samples to one of the given classes and identified informative combinations of transcripts. The high informative power of a combination of transcripts was defined as high reliability of the classifier based on this combination. Classifiers were constructed using Support Vector Machine (SVM), more specifically soft-margin SVM^10^ with linear kernel.

A microarray dataset consisting of ER-positive breast cancer samples was taken for the examination of the proposed hypothesis that genes with low level of differential expression can in combinations nevertheless reliably reveal phenotypical differences. Validation procedure used a similar dataset as well as an RNAseq-based dataset.

## Results

### Outline of the experiment

A key step in the present study is the analysis of the informative power of all pairs of genes. The samples were divided into three non-overlapping groups including training group, filtration group (consisting of two sub-groups) and validation (testing) group. The training group comprised microarray data from GSE17705 dataset^11^, the filtration sub-groups contained microarray datasets GSE12093^12^ and GSE6532^13^, and the dataset GSE3494^14^ was used for validation. The numbers of patients in each group are indicated in Supplementary Table 2.

Genes with low or constant expression level in the training dataset were excluded from the analysis (see Methods). The analyzed set consisted of 14,712 probesets corresponded to 10,060 genes.

For each pair of probesets a binary classifier was constructed separating the groups of patients with recurrence within five years after surgery and no recurrence. We used the training dataset and the support vector machine approach (soft-margin SVM with linear kernel) in this step. The reliability of the classifier was evaluated on the training group as well as on the two filtration subgroups. If the quality of the classification met the accepted criteria, the pair was considered informative and was further used on the validation dataset. Filtration based on additional groups along with the training dataset provided the removal of gene pairs that worked well for the training dataset solely because of the specific properties of the training dataset or as a result of a coincidence due to the large number of analyzed classifiers (more than 100 million).

**Figure 2:**
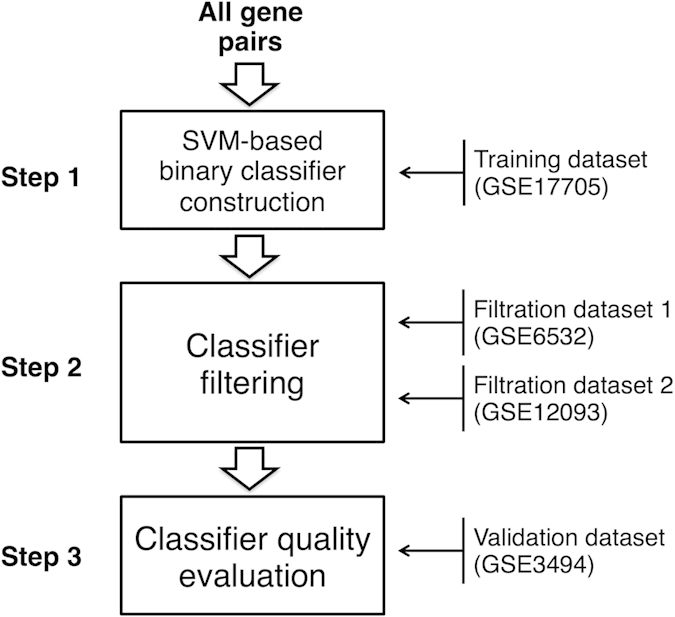
Scheme of gene pairs analysis.

The general outline of the analysis of gene pairs is shown in Fig. 2.

This analysis was followed by the construction of larger gene combinations performed by a specially developed greedy-type extension algorithm.

The total amount of computation required for the construction of gene pairs and larger gene combinations exceeded 50,000 CPU-hours.

### AUC-based study of gene pairs

The area under the ROC-curve (AUC) is a commonly accepted characteristic of the quality of the classifier. For a random sample attribution AUC equals 0.5, and error-free classifiers have AUC equal to 1. We set the AUC threshold to 0.75 for the training dataset and to 0.7 for each filtration dataset. This resulted in 570 informative probeset pairs out of more than 100 million initial pairs (Supplementary File) which corresponded to 547 unique pairs of genes.

The resulted mean value of AUC for the validation group was 0.662 (95-percent confidence interval for the mean (0.659, 0.665), median 0.663). We tested a null hypothesis that the mean value of AUCs of the informative classifiers for the validation group was 0.5, i.e. corresponded to the reliability of a random classification. The p-value of this hypothesis was less than 2.2 × 10^−15^ confirming the reliability of constructed classifiers.

Notably, the classifiers comprised 343 genes in total with some genes belonging to more than 20 classifiers (Table 1).

Most of these genes are known to be biomarkers of breast cancer or involved in cancer progression, including SQLE^15^, CTTN^16^,^17^, TTK^18^,^19^, RACGAP1^20^,^21^, KIF4A^22^, and CCNA2^23^. However for the gene TOMM70A there was no evidence for any role in cancer so far.

We performed category enrichment analysis for all 343 genes (Supplementary Table 1). The top of revealed categories includes phosphoproteins, acetylation, regulation of cell cycle and cell division all known to be highly relevant to malignant progression.

Interestingly, the majority of probesets forming informative pairs had low degree of differential expression with respect to 5-years recurrence and recurrence-free patients from the training dataset. More specifically, for the fold change threshold of 1.5× the number of probesets did not exceed 7% (23 probesets corresponding to 22 genes), whereas the total number of probesets having such fold change was 67 (60 genes). Thus the traditional approach focused on the genes having significant differential expression would exclude the majority of genes forming the informative pairs out of the researcher’s focus.

Remarkably, Table 1 contains only one gene with fold change higher than 1.5×, namely, SQLE. Moreover, nearly 90% of informative pairs consisted of probesets having the fold change lower than 1.5×, and there were no pairs having both probesets of higher fold change. Finally, the reliability of classification by the pairs with one probeset of fold change ≥1.5× was equal to the reliability provided by pairs without such probesets (t-test two-tailed p-value 0.276).

The AUC mean values for the training and filtration datasets and AUC values for the validation dataset correlated significantly with the Spearman’s rank correlation p-value 4.4 × 10^−12^ which means that higher AUC mean value for training and filtration datasets consistently corresponded to a higher AUC for the validation dataset (Supplementary Fig. 1). Thus the AUC mean value for the training and filtration datasets is itself an important parameter predicting the classification quality for another dataset and can be used as a criterion to rank the identified pairs of genes.

### Additional sensitivity- and specificity-based filtering of gene pairs

Even in cases of high AUC values using the same classifier threshold for different datasets can be impossible or may result in a bias towards high sensitivity in combination with low specificity or the opposite bias (high specificity with low sensitivity) depending on datasets. In order to achieve balanced classification results using the same threshold we additionally performed filtration of identified gene pairs with the thresholds for the sensitivity and specificity. More specifically, for each parameter the threshold was set to 0.65 for all datasets. This filtration resulted in 14 pairs (Table 2) having the AUC mean value 0.697 (median 0.698) and mean values of sensitivity 71.0% (median 72.7%) and specificity 60.7% (median 62.1%) for the validation dataset. Remarkably, there was only one pair containing a probeset with fold change ≥1.5×, namely PRC1 in the pair of DLG3 and PRC1.

**Figure 3:**
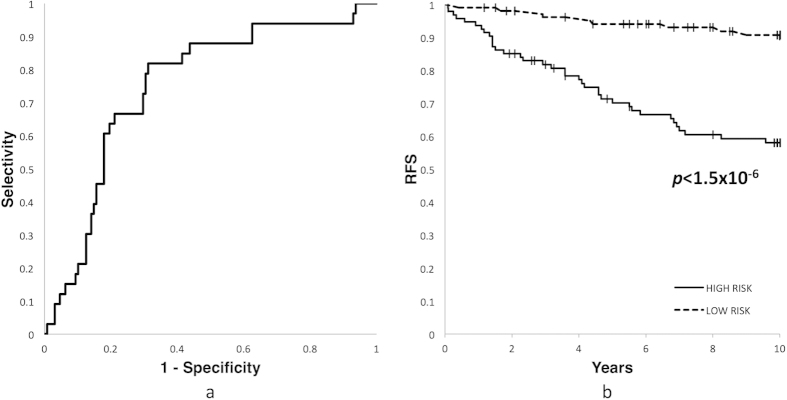
The properties of the classifier based on the gene pair IGFBP6 and ELOVL5 for the validation dataset. (**a**) ROC-curve. (**b**) Kaplan-Meier curves. RFS – recurrence free survival.

The pair IGFBP6-ELOVL5 showed the highest AUC mean value (0.765) for the training and filtration datasets. When the same coefficients and threshold were used, this pair exhibited outstanding classification quality for the validation dataset as well (AUC 0.749, sensitivity 81.8% and specificity 62.5%) (Fig. 3). This remarkable reliability of the classification is similar^24^ to the one provided by well-known gene signatures such as OncotypeDX and MammaPrint^6^, which however include more genes (21 and 70, respectively).

**Figure 4:**
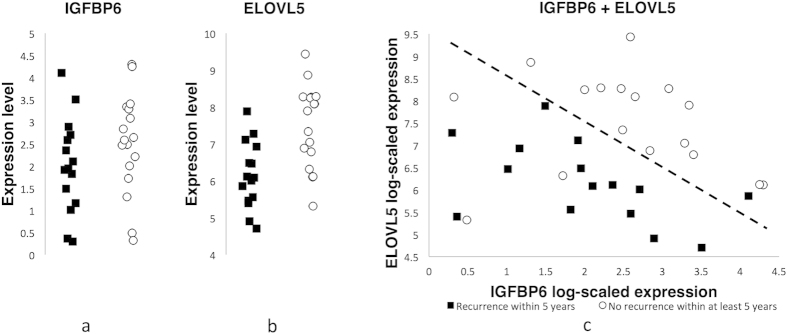
The expression levels of IGFBP6 and ELOVL5 for patients with and without recurrence measured by RNA-sequencing. (**a**) Log-scaled expression of IGFPB6. (**b**) Log-scaled expression of ELOVL5. (**c**) The joint distribution of expressions of IGFBP6 and ELOVL5.

We tested the IGFBP6-ELOVL5 classifier on the breast cancer dataset from TCGA Research Network (http://cancergenome.nih.gov/) obtained using RNA-seq. Surprisingly, in spite of completely different technologies of microarrays and sequencing, both sensitivity and specificity of the classifier exceeded 80% (Fig. 4). This observation points out to the fact that classifiers generated by the presented approach may be universal and can be applied to any group of ER-positive breast cancer samples even if they have been obtained using other technologies for gene expression analysis.

### The construction of larger combinations of genes

We investigated whether the classification quality can be improved by increasing the number of genes in the analyzed gene combinations. Since the exhaustive analysis for gene triples would already need more than 2,5 million CPU-hours and is computationally infeasible, we developed an alternative two-step approach. First we analyzed all possible extensions of 570 previously identified informative pairs by one out of the 14,712 probesets and filtered out the triples which did not pass AUC, sensitivity and specificity thresholds for training and filtration datasets. The second step consisted in a greedy element-wise optimization. The optimization started by the analysis of all possible changes of the first element in a triple and selection of the change resulting in the maximal AUC mean value for the training and filtration datasets and passing the additional thresholds on sensitivity and specificity. Then the same procedure was performed for the second and the third element of the triple and then again for the first one and so on until there was no further increase of the AUC mean value. A similar approach was used to construct quadruples starting from the triples and quintuples starting from the quadruples.

We obtained 426 triples, 291 quadruples and 414 quintuples. As expected, the increase of the number of genes in a classifier resulted in a more reliable classification (Supplementary Fig. 2a). More specifically, the transition from pairs to triples was accompanied by the increase of the AUC median value for the validation dataset from 0.663 to 0.669 (two-tailed U-test p-value 1.7 × 10^−6^) and the AUC mean value from 0.662 to 0.679. The transition from triples to quadruples consistently resulted in further increase of the AUC median value up to 0.694 (two-tailed U-test p-value when comparing with triples 3.9 × 10^−5^) and the AUC mean value up to 0.690. During the transition from quadruples to quintuples we observed saturation or even overtraining, namely the increase of AUC on the training dataset did not lead to the similar change of this value on the validation dataset. We even found a decrease of AUC value for the validation dataset comparing to the quadruples and triples (AUC median value down to 0.669 and AUC mean value down to 0.670). At the same time, the transition to quintuples led to a significant decrease of AUC deviation (Supplementary Fig. 2b).

Interestingly, we directly compared this greedy approach with the exhaustive analysis described above and found that the exhaustive analysis had significantly higher efficiency for gene pairs (Supplementary Text).

## Discussion

Genome-wide expression analysis is a common tool, which is routinely used nowadays to identify genes which are associated with disease. It is accepted by definition to consider only the most differentially expressed so-called «top-genes» when comparing expression profiles of sample groups^25^,^26^,^27^. However this traditional approach omits the majority of yet moderately differentially expressed genes completely. The approach presented here proves that these genes can contribute significantly to the construction of highly informative gene signatures.

We have compared SVM-based exhaustive search and a greedy-type extension algorithm and found that the gene signatures with the highest informative power are identified by the exhaustive search. The limitation of this method is the necessity of the use of a supercomputing facility. However, the significance of this limitation will probably be reduced due to the technical progress within the next years. The greedy-type extension algorithm is also resource-consuming, but it still allows construction of larger gene signatures in comparison with the exhaustive search construction.

We investigated the number of genes comprising the signatures and found that the informative power increases during the transition from 2 to 3 and then to 4 genes in a single signature. Further increase was diminished which can be explained by over-training of the classifier. This observation suggests that the optimal number of genes in an informative signature is limited to 3 or 4 providing a solid basis for the identification of pathways or gene cascades potentially involved in the biological processes of interest.

The reported approach identified highly informative gene signatures (pairs) using microarray expression datasets, and these signatures turned out to work efficiently on RNA sequencing-based dataset of ER-positive breast cancer patients. This points out to the fact that the classifiers constructed using our method may be universal and applicable to any other datasets of ER-positive breast cancer patients irrespectively of the gene expression analysis platform used.

The reported method allows to go beyond the limitation of the “top-genes” and covers the majority of analyzed genes. This broad approach could explain the robustness of gene signatures^28^ identified by our method and the observed inconsistencies of many marker genes claimed to be of prognostic or predictive value for cancer patients in other reports.

We suppose that the presented approach can potentially be applied not only for RNA expression datasets but for any other kind of biological high-throughput data including metabolome and proteome and can be adapted to SNP and methylome datasets.

## Methods

### Microarray datasets

All analyzed microarray datasets used Affymetrix U133A platform. Raw data (CEL files) corresponding to patients with ER-positive breast cancer and the known recurrence status were downloaded from Gene Expression Omnibus (GEO) and jointly preprocessed using RMA method^29^. The pre-processing used RMA Bioconductor^30^ package xps.

The pre-processed set included samples from the GSE17705^11^ dataset used for training, datasets GSE6532^13^ and GSE12093^12^ used for filtration, and GSE3494^14^ used for validation. The number of ER-positive samples with known recurrence status for these datasets is presented in Supplementary Table 2.

### Sequencing dataset

The RNASeq data and clinical annotations were downloaded from TCGA BRCA databank^31^. Gene-level expression data for ER-positive patients (er_status_by_ihc = Positive) with no recurrence (vital_status = Alive, tumor_status = TUMOR FREE) within at least 7 years after surgery and ER-positive patients with death (vital_status = Dead, tumor_status = WITH TUMOR) within 5 years after surgery were extracted from UNC IlluminaHiSeq_RNASeq 01A-datafiles. The data were available for 17 patients without recurrence and 15 patients with death. RPKM (Reads Per Kilobase per Million mapped reads) value was used as an expression level characteristic^32^.

### Classification reliability parameters

The standard definitions of classification reliability parameters were used. The sensitivity was defined as the ratio of the number of correctly predicted recurrences (tp) to the total number of samples corresponding to patients with recurrence (i.e., the sum of (tp + fn)). The specificity was defined as the ratio of correct predictions for recurrence-free patients (tn) to the total number of samples corresponding to recurrence-free patients (i.e., the sum of (tn + fp)). For measuring the sensitivity and specificity as percentage, corresponding ratio was multiplied by 100%.

The ROC-curves were constructed as graphs of the parametric dependence of false positive rate (equal to (1 - specificity), plotted against the X-axis) and sensitivity (plotted against the Y-axis) on the classifier threshold which varied from −∞ to +∞. AUC was defined as the area between the X-axis and the ROC-curve. Since the ROC-curve is a polygonal line consisting of vertical and horizontal segments the area calculation was performed using a straightforward decomposition of the area under the ROC-curve into non-overlapping rectangles and summation of areas of these rectangles.

The calculation of sensitivity, specificity, and AUC included only the samples corresponding to patients with recurrence within the first five years after surgery and patients without recurrence within at least 7 years. The remaining samples comprising a “gray zone” were not considered.

Kaplan-Meier curves were plotted using all samples, including the “gray” zone.

### The filtration of probesets

The filtration of probesets was based solely on the training dataset with the excluded “gray zone” samples. Probesets without associated Gene Symbols were excluded from the analysis along with probesets with constantly low expression (expression level less than 128, i.e., log-scale 7, for all samples) and probesets with low changes in expression (the ratio of maximum expression and minimum expression in standard scale lower than 2).

### The construction of classifiers

Classifier construction utilized soft-margin Support Vector Machine (SVM) approach^10^ with a linear kernel and penalty weights inversely proportional to the number of samples in each class. Within the construction, three different values of penalty factor *C* were taken: 1, 16 and 256. These values were determined by cross-validation in the preliminary analysis of random combinations of genes. If thresholds for AUC, sensitivity and specificity were met for two or three values of *C*, the classifier which provided a higher mean value of AUC for the training and filtration datasets was chosen.

The classifier construction used log-scaled expression levels. Prior to the SVM utilization log-scaled expression levels for each probeset were linearly normalized: the mean expression level of the probeset was subtracted and then the values were divided by the standard deviation of the expression level of the probeset. The computation of means and standard deviations used only data from the training dataset with the excluded “gray zone” data.

The constructed classifiers were applied to the filtration and validation datasets without any changes in coefficients, threshold or normalization parameters (shifts and contraction coefficients).

### Thresholds used

Thresholds used for the selection of informative gene combinations are summarized in Supplementary Table 3. These thresholds provided comparable numbers of the selected pairs, triples, quadruples and quintuples, which had an order of several hundred.

### Statistical analyses

To identify the relationship between the variables Spearman’s rank correlation coefficient was used. Means for normally distributed variables were compared using Student’s two-sample t-test. Comparisons of means of normally distributed variables with a specified value was performed using Student’s one-sample t-test. Variables with non-normal distribution were compared using Mann-Whitney U-test. The normality check was performed using the Shapiro-Wilks test. The calculation of confidence intervals for means of normally distributed variables was based on quantiles of Student’s t-distribution. The statistical analysis of Kaplan-Meier curves used the log-rank test. Category enrichment analysis was performed using DAVID^33^.

### Software implementation

The software implementation of the described method was performed in C/C++ programming language. It used libsvm^34^ implementation of SVM. The data-level MPI-based parallelization was done for efficient employment of supercomputing resources.

## Additional Information

**How to cite this article**: Galatenko, V. V. *et al*. Highly informative marker sets consisting of genes with low individual degree of differential expression. *Sci. Rep*. **5**, 14967; doi: 10.1038/srep14967 (2015).

## Supplementary Material

Supplementary Information

Supplementary Datasets

## Figures and Tables

**Figure 1:**
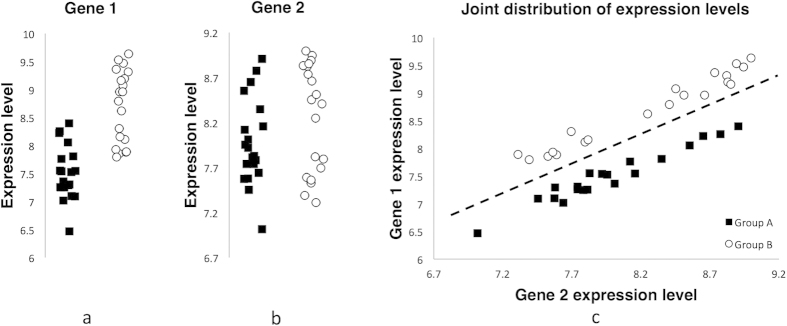
Synthetic example of a contribution of a gene with a low degree of differential expression into the cumulative informative power of the pair of genes. (**a**) Expression of gene 1 in Groups A and B; (**b**) expression of gene 2 in Groups A and B; (**c**) the joint distribution of the expression of genes 1 and 2 in Groups A and B.

**Table 1:** Genes included in at least 20 informative gene pairs (out of 547).

Gene Symbol	Number of informative pairs
SQLE	109
DSCC1	85
CTTN	43
TOMM70A	37
TTK	34
RACGAP1	29
ELOVL5	29
KIF4A	27
CCNA2	23

**Table 2:** Informative gene pairs that satisfied the additional constraints the sensitivity and specificity.

Gene Symbol 1	Gene Symbol 2	AUC	Sensitivity	Specificity
IGFBP6	ELOVL5	0.749	81.8%	62.5%
HSPD1	ELOVL5	0.756	72.7%	62.5%
TTK	CADPS2	0.721	78.8%	57.8%
RUNX1	SQLE	0.645	72.7%	51.6%
ELOVL5	PPIA	0.721	81.8%	56.3%
PSMD2	TTK	0.679	66.7%	59.3%
LGR4	KIF4A	0.678	69.7%	64.8%
DCTD	SQLE	0.635	54.5%	65.6%
ELP4	KIF4A	0.717	78.8%	65.6%
BTN3A3	RACGAP1	0.668	60.6%	71.9%
TTK	DIRAS3	0.745	75.8%	61.7%
HSPD1	IL6ST	0.733	75.8%	62.5%
REST	SQLE	0.637	63.6%	52.3%
DLG3	PRC1	0.664	60.6%	54.7%

AUC values, sensitivity and specificity are shown for the validation dataset.
