# Supplementary material for: Highly informative marker sets consisting of genes with low individual degree of differential expression
Source: Sci Rep. 2015 Oct 8;5:14967. doi: 10.1038/srep14967 (PMC4597361; doi:10.1038/srep14967)
Supplement: Supplementary Information [file srep14967-s1.doc]

***Highly informative marker sets consisting of genes
with low individual degree of differential expression***

***V.V. Galatenko, M.Yu. Shkurnikov, T.R. Samatov, A.V. Galatenko, I.A. Mityakina, U. Schumacher, A.G. Tonevitsky***

***SUPPLEMENTARY INFORMATION***

***Supplementary Tables***

Supplementary Table 1 | Categories over-represented in the list of genes included in the informative pairs.

| **Term** | **Count** | **Adj.p-value** |
| --- | --- | --- |
| phosphoprotein | 189 | 4.2x10^-10^ |
| acetylation | 89 | 5.7x10^-8^ |
| cell cycle | 31 | 5.9x10^-8^ |
| mitotic cell cycle | 29 | 2.3x10^-6^ |
| M phase | 26 | 4.3x10^-6^ |
| cell division | 20 | 1.6x10^-5^ |
| chromosome segregation | 13 | 2.0x10^-5^ |
| mitosis | 20 | 2.2x10^-5^ |
| nuclear division | 20 | 2.2x10^-5^ |
| organelle fission | 20 | 3.2x10^-5^ |
| microtubule cytoskeleton | 32 | 5.0x10^-5^ |
| regulation of cell cycle | 22 | 4.2x10^-4^ |
| cytoskeleton | 50 | 2.1x10^-3^ |
| Apoptosis | 19 | 4.7x10^-3^ |

Supplementary Table 2 | The number of samples in the utilized datasets.

| **Dataset accesion** | **Dataset type** | **Patients** | | |
| --- | --- | --- | --- | --- |
|  |  | **Total number** | **Recurrence within 5 years** | **Without recurrence for at least 7 years** |
| GSE17705 | Training | 298 | 42 | 159 |
| GSE6532 | Filtration | 196 | 49 | 76 |
| GSE12093 | Filtration | 136 | 12 | 67 |
| GSE3494 | Validation | 201 | 33 | 128 |

Supplementary Table 3 | Thresholds used for the selection of informative gene combinations.

| **Combination size** | **AUC (training / filtration)** | **Sensitivity (training and filtration)** | **Specificity (training and filtration)** |
| --- | --- | --- | --- |
| Pairs | 0.75 / 0.70 | 0.65 | 0.65 |
| Triples | 0.78 / 0.73 | 0.68 | 0.68 |
| Quadruples | 0.81 / 0.78 | 0.71 | 0.71 |
| Quintuples | 0.84 / 0.81 | 0.75 | 0.75 |

***Supplementary Figures***

***
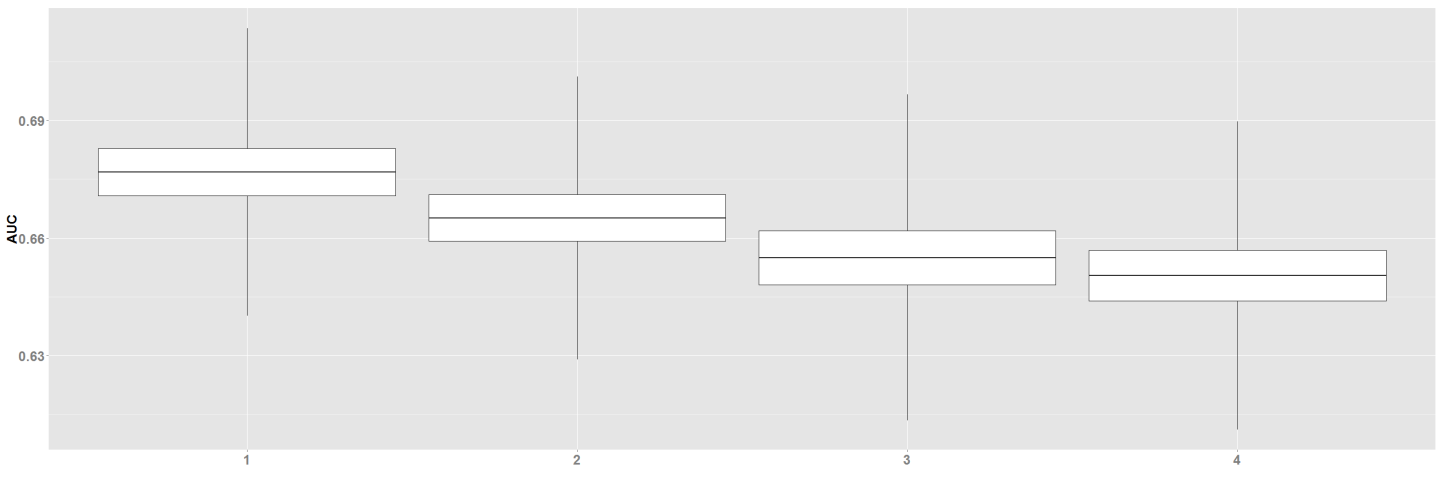
***

Supplementary Figure 1 | Increase in classification reliability for the validation dataset with an increase in mean value of AUC for the training and filtration datasets. The set of 570 informative classifiers was ranked by the mean value of AUC for the training and filtration datasets and divided into four equal-size groups (quarters). For each quarter the mean value of AUC for the validation dataset is shown (horizontal line) along with 95% confidence interval for the mean value of AUC (rectangle) and mean ± sd intervals (vertical lines). Quarters are arranged from left to right in a descending order with respect to the mean value of AUC for the training and filtration datasets. For the validation dataset the mean value of AUC for the first quarter was significantly higher than the mean value of AUC for the second two-tailed t-test p-value 0.007), and similarly the mean value of AUC for the second quarter was significantly higher than the mean value of AUC for the third quarter (two-tailed t-test p-value 0.029).

***
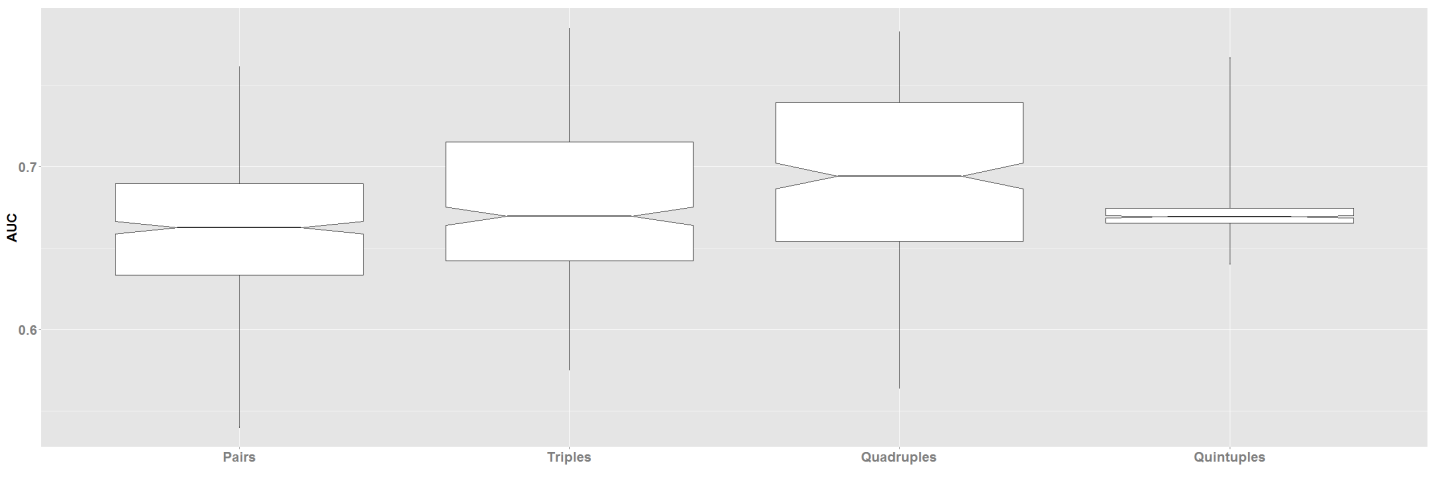
***

**a**

***
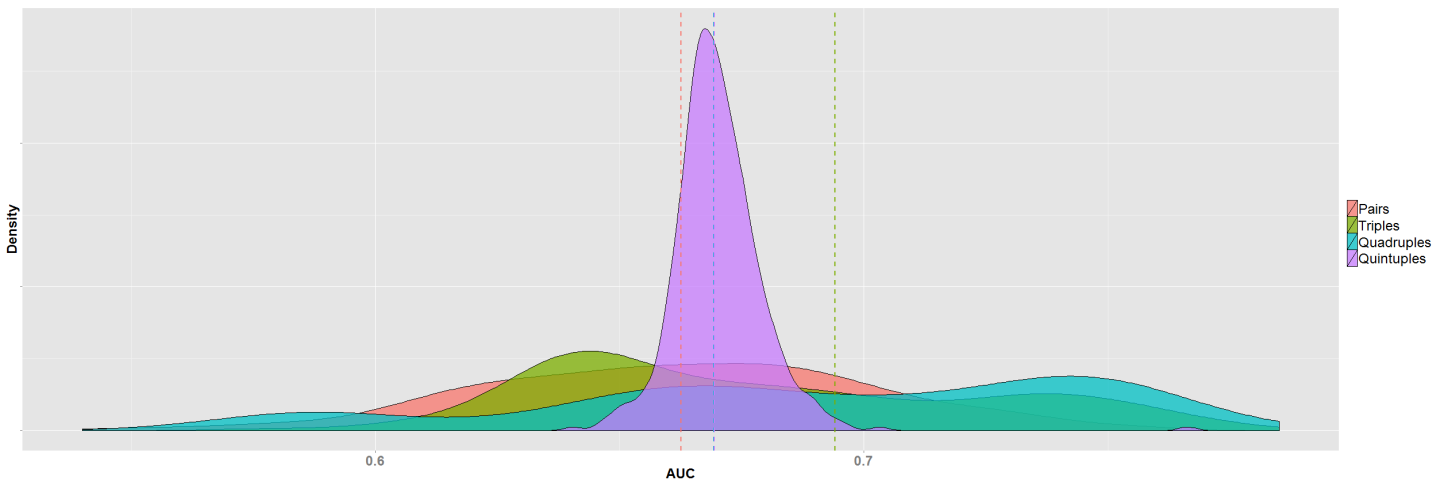
***

b

Supplementary Figure 2 | Changes in the classification quality (measured by AUC) for the validation dataset with an increase in the number of genes in combinations. a) Notched box plots: horizontal lines show median values, notches show median confidence intervals (median ± 1.57 x InterQuartileRange / SampleSize^1/2^), boxes show interquartile ranges and vertical lines show ranges between minimum and maximum values. b) The distribution density of AUC.

***Supplementary Text***

In order to compare the exhaustive analysis of classifiers and the construction of classifiers based on the greedy-type extension algorithm, we applied the greedy-type extension algorithm for the construction of informative gene pairs.

First, we constructed pairs starting from all 67 probesets with fold change exceeding 1.5x for the training dataset. As thresholds used for additional filtration of pairs in case of exhaustive analysis were extremely hard for both extension and element-wise optimization stages (only 14 pairs in total satisfied that constraints), we uniformly decreased thresholds. Namely, we set thresholds 0.63 for sensitivity and specificity on the training and filtration datasets, 0.73 for AUC on the training dataset and 0.68 for AUC on the filtration datasets.

The extension stage of the construction resulted in a set of 57 gene pairs. Greedy-type element-wise optimization of this resulting set led to 10 gene pairs – four pairs which were already present in this set and six new. So totally the greedy-type extension algorithm constructed 63 gene pairs. Interestingly, the output of the optimization stage provided higher classification quality in comparison with the output of the extension stage (mean AUC for the validation dataset 0.69 and 0.66 respectively; two-tailed t-test p-value 0.03).

Only three pairs among the constructed 63 satisfied the constraints used in exhaustive analysis for additional filtration of gene pairs, while the total number of such gene pairs (as identified by the exhaustive analysis) was 14. One of these three gene pairs was constructed by the extension stage, and two others were constructed by the greedy-type element-wise optimization.

The comparison of AUC values on the validation dataset showed that the classification quality provided by 63 gene pairs constructed by the greedy-type extension algorithm and by 570 gene pairs that passed AUC-based filtration were similar (in both cases mean value of AUC was 0.66). However, 14 pairs identified by the exhaustive search that satisfied the additional constraints on sensitivity and specificity significantly outperformed 63 gene pairs constructed by the greedy-type extension algorithm (for these 14 pairs the mean value of AUC was 0.70; two-tailed t-test p-value 0.007).

Furthermore, we performed a similar construction of pairs starting from a set of random genes with the same size (67 genes). In this case the extension step resulted only in only one pair, which however provided high classification quality (mean AUC for the training and filtration datasets 0.73, AUC for the validation dataset 0.74) and was absent in the list of 63 pairs that was constructed in case of starting from probesets with fold change exceeding 1.5x. At the same time, the greedy-type element-wise optimization of this pair resulted in a pair with similar classification quality (mean AUC for the training and filtration datasets 0.77, AUC for the validation dataset 0.73) which was present in the list of 63 pairs.

We also performed gene pair construction staring from an empty set. The first element was selected by a greedy optimization of mean value of AUC for the training and filtration datasets without any additional constraints. The second element was selected by a similar greedy optimization but with constraints on sensitivity and specificity (at least 0.63 for the training and filtration datasets) and AUC (at least 0.73 for the training dataset and at least 0.68 for the filtration datasets). Then greedy-type element-wise optimization with the same constraints was applied. The resulting gene pair belonged to the list of 63 pairs that was constructed in case of starting from probesets with fold change at least 1.5x.

Thus, greedy-type extension algorithm provided the construction of reliable classifiers and required essentially lower amount of computation in comparison with the exhaustive analysis. However, the amount of computation was still considerable (for the constructions above – more than 70 CPU-hours), and most of the informative gene pairs identified by the exhaustive analysis were missed by this extension algorithm even when starting from a set of genes with the highest fold changes. Starting from random sets provided the construction of only very small number of informative gene pairs. Interestingly, the greedy-type element-wise optimization stage of the extension algorithm required more computation, but provided construction of new combinations with significantly higher classification reliabilities in comparison with the starting points.
